# Kinesthetic Feedback for Understanding Program Execution

**DOI:** 10.3390/s23115159

**Published:** 2023-05-29

**Authors:** Satinder Gill, Bryson J. Goolsby, Dianne T. V. Pawluk

**Affiliations:** Department of Biomedical Engineering, Virginia Commonwealth University, Richmond, VA 23219, USA; goolsbybj@vcu.edu (B.J.G.); dtpawluk@vcu.edu (D.T.V.P.)

**Keywords:** programing, scratch, robotics, visually impaired users

## Abstract

To better prepare future generations, knowledge about computers and programming are one of the many skills that are part of almost all Science, Technology, Engineering, and Mathematic programs; however, teaching and learning programming is a complex task that is generally considered difficult by students and teachers alike. One approach to engage and inspire students from a variety of backgrounds is the use of educational robots. Unfortunately, previous research presents mixed results on the effectiveness of educational robots on student learning. One possibility for this lack of clarity may be because students have a wide variety of styles of learning. It is possible that the use of kinesthetic feedback, in addition to the normally used visual feedback, may improve learning with educational robots by providing a richer, multi-modal experience that may appeal to a larger number of students with different learning styles. It is also possible, however, that the addition of kinesthetic feedback, and how it may interfere with the visual feedback, may decrease a student’s ability to interpret the program commands being executed by a robot, which is critical for program debugging. In this work, we investigated whether human participants were able to accurately determine a sequence of program commands performed by a robot when both kinesthetic and visual feedback were being used together. Command recall and end point location determination were compared to the typically used visual-only method, as well as a narrative description. Results from 10 sighted participants indicated that individuals were able to accurately determine a sequence of movement commands and their magnitude when using combined kinesthetic + visual feedback. Participants’ recall accuracy of program commands was actually better with kinesthetic + visual feedback than just visual feedback. Although the recall accuracy was even better with the narrative description, this was primarily due to participants confusing an absolute rotation command with a relative rotation command with the kinesthetic + visual feedback. Participants’ zone location accuracy of the end point after a command was executed was significantly better for both the kinesthetic + visual feedback and narrative methods compared to the visual-only method. Together, these results suggest that the use of both kinesthetic + visual feedback improves an individual’s ability to interpret program commands, rather than decreases it.

## 1. Introduction

Science, Technology, Engineering and Mathematics have a significant impact on the economy, with two out of three jobs in the United States and nearly 70% of the nation’s GDP attributed to activities in these fields [1]. In addition, the projected growth in these fields is expected to be over two times faster (8%) than in other areas [2]. Computer programming is an important part of STEM, both as its own discipline and as an integral component of other STEM fields [3,4,5]. Additionally, research has shown that learning how to program contributes to the development of other higher-level skills, such as problem-solving, inferencing, creative thinking and even language skills [6,7,8,9].

However, teaching and learning programming is a complex task that is generally considered difficult by students and teachers alike [10,11,12]. As an example, the average programming ability score of first-year CS students is reported to be only about 23.89 out of 110 [13]. Additionally, high attrition rates (30–40%) in early programming courses indicate how students struggle with programming [14]. A variety of approaches to teaching programming have been proposed to improve engagement and learning, such as the use of games, visual programming, collaborative work, pair programming, a read before write approach, etc. [15,16,17,18,19,20,21]. One approach that has exploded in the past two decades has been the use of educational robots [22].

Educational robots have inspired students from diverse backgrounds, in terms of ethnicity, culture, gender and socio-economic status, to become interested in learning computer science. Robots instinctively spark interest and curiosity in children [23]. Research has also shown that educational robots contribute more to a student’s emotional and intellectual engagement than other commonly used educational tools, due to the physical embodiment of the robotic kits and the possibility of promoting students’ communication skills [24].

The foundation for the believed effectiveness of educational robots in student learning is based on the theory of constructionism, in which a student’s construction of new knowledge is built on their prior knowledge through their interaction with the environment. The emphasis is on discovery learning with tangible objects that fuels the constructive process [22,23]. However, research results on the effectiveness of educational robots on student learning have been mixed [22,25]. For example, the results presented in [26,27] support the idea that robots are effective as a teaching tool for programming whereas the results presented in [28] reported otherwise. Additionally, some studies report that robots are effective in increasing problem-solving skills [29,30], whereas others have reported the contrary [31,32]; therefore, there is a clear need to investigate this further.

One possibility for this lack of clarity may be because students are not a monolithic group when it comes to learning. Educational researchers have observed that different learners often have a differential preference for one learning style over another, and that this affects the outcome of the learning process. One significant factor that is recognized is the perceptual preference in learning media such as the visual (graphical), aural, read/write and kinesthetic modalities given in the VARK model [33]. Although the current description of kinesthetic preference is a preference for the use of experience and practice in learning, whether simulated or real, it may also be important to consider the direct meaning of the word in terms of the use of kinesthetic movement/sensation and haptics (i.e., touch + kinesthetic sensation). Relatively recently, several studies [34,35] found a benefit of using kinesthetic movement/sensation in a multisensory approach to learning. These studies found that learners improved their understanding of graphs and diagrams by finger tracing the data/information. Previous work [36] also found that the use of haptic feedback in combination with finger tracing increased young students’ understanding of the alphabet.

It is possible that the use of kinesthetic feedback, in addition to the normally used visual feedback, could increase the benefit of educational robots to a larger number of students by encompassing a larger number of learning styles; however, this is predicated on the assumption that the addition of kinesthetic feedback will not decrease a student’s integrated, multisensory perception of the sequence of commands executed by the robot nor the magnitude of their parameters. This information is essential to determining whether a program is working correctly and for debugging purposes. Unfortunately, there is some indication [37] that kinesthetic memory does not provide as spatially-accurate information as visual information. It is currently unclear as to the resulting accuracy of the integrated, multisensory percept, especially since any method of kinesthetically following a robot will occlude a student’s vision.

The objective of the work described in this paper was to determine whether individuals are able to accurately determine a sequence of movement commands and their magnitude when using both kinesthetic and visual feedback. The particular kinesthetic/haptic feedback considered was from students’ resting one of their hands on a tabletop mobile robot. This was contrasted with the use of only visual feedback as a “control” condition. For the visual feedback condition, we used the Scratch visual stage where the program was executed as an animation of a cartoon cat. We undertook this for a few reasons. First, Scratch is currently ubiquitous in K-12 computer science classrooms, as well as being used in some university introductory programming courses. Second, previous research has suggested that there is no difference on student performance whether physical or simulated robots are visually observed [38]. Third, using a simulated robot is more cost-effective. We did not consider kinesthetic/haptics alone, as there is evidence to suggest that haptics without vision is detrimental to the learning process [39].

## 2. Materials and Methods

### 2.1. Experimental Protocol and System Overview

Ten individuals (three male and seven female, aged 19–57 years, with a mean ± SD = 23.0 ± 13.9 years) with no known vision impairments participated in this study. All the subjects provided written informed consent before participation. This study was approved by the Virginia Commonwealth University Internal Review Board (HM20020574).

The study participants were given three different feedback conditions, with their order of presentation counterbalanced across participants. The first condition was kinesthetic + visual, which was generated using a tabletop, physical, mobile robot modified to provide a handle for a user to rest their hand. The second condition was visual only, which was generated on the Scratch visual stage in its online environment using the default cat sprite. A third, auditory condition, was added for comparison, which provided a narrative of the program commands executed.

Ten trials were given in a block for each condition. For each trial, the study participants were presented with the execution of a computer program consisting of a sequence of movement commands in the given feedback mode. The movement of the mobile robot/sprite was described by both an x, y location and a direction that it faced. The movement commands were drawn from the list given in Table 1. The number of commands in a given computer program sequence ranged from 2 to 4. Three sets of ten movement sequences were generated so that all trials were unique programs. The movement commands and their parameter values were generated randomly from within their given ranges. Each of the sets of ten movement sequences was assigned randomly to a given condition for each participant to minimize any bias due to the set difficulty.

After experiencing the robot/sprite movement, the participants were asked to state the movement commands in the sequence and the planar location of the robot/sprite after each command. As participants in a pilot study found it very frustrating to provide x, y coordinates, the participants in the actual study were only asked to provide the location in terms of one of nine zones in the robot workspace (Table 2).

Before the testing began for each condition, the participants received training on that mode of presentation. First, the experimenter explained to the participant how the program information was to be presented to them and what pieces of information they needed to provide to the experimenter at the end of the program execution. Next, a practice session was carried out using a set of trials not part of the testing, to familiarize the participants with the testing modality. The experiment only proceeded once a participant felt confident about their understanding of the method. The same process was repeated for each of the three feedback modalities.

After each condition was completed, the participants were given a questionnaire similar to the System Usability Scale (Table 3; Brooke, 1995 SUS: A quick and dirty usability scale). Each usability quality was given in both its positive and negative form to reduce bias. The participants were asked to give a score on a Likert scale (where 1—strongly disagree and 5—strongly agree) for the questions given in Table 3.

Lastly, participants were asked to rank their preference of the three feedback modalities for the following set of questions.

### 2.2. Narrative

In the auditory (narrative) mode, the experimenter spoke the sequence of commands executed out loud, as well as the planar location of the robot/sprite at the end of each command, to the participant. The participants were allowed to ask the experimenter to repeat the narrative until they felt confident about their understanding of the program. They were then provided with only a single attempt to accurately provide the sequence of commands, and their end points, to the experimenter verbally. The user response information was recorded using a Microsoft Excel worksheet for later processing.

### 2.3. Visual Only (Computer Animation)

**Figure 1:**
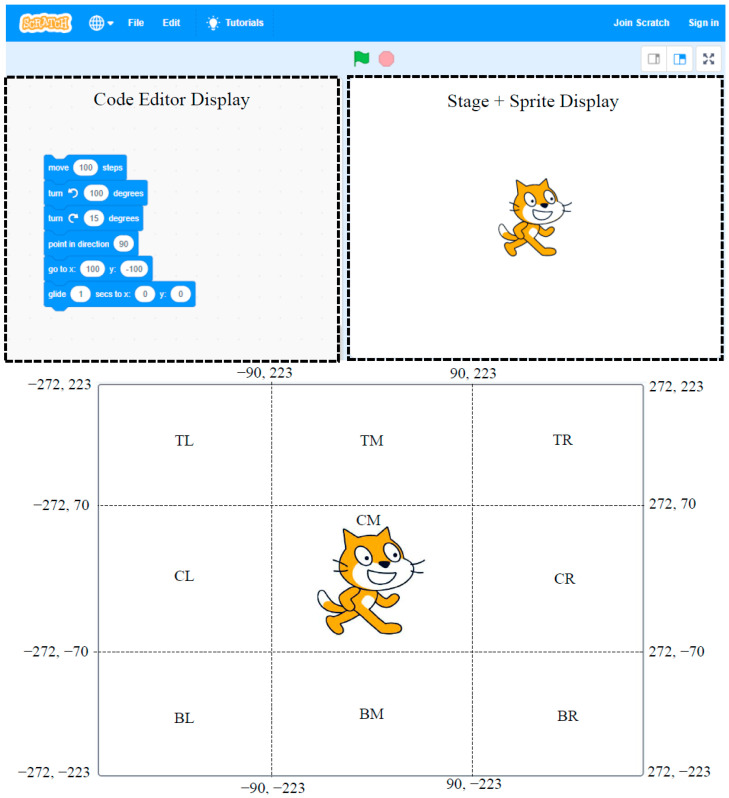
(**Top**): Scratch program “code editor” and “stage + sprite” display; (**Bottom**): division of “stage + sprite” display into different zones based on Table 2.

This method consisted of using the block-based visual programming language called Scratch. The Scratch display can be primarily divided into two displays: the “code editor” display and the “stage + sprite” display (Figure 1). In this experiment, both displays were visible to the experimenter whereas the participant sat in front of a computer screen with a “stage + sprite” display only. For each trial, the experimenter implemented a sequence of commands using the code editor. The result of the program implementation was displayed to the participant in the form of sprite movement on the stage. Similar to the narrative method, the participants were allowed to request a repetition of the sprite movement sequence until they felt confident about their understanding of the program. They were only allowed a single attempt to repeat a sequence of commands back to the experimenter. This information (i.e., the sequence of instructions and planar positions) was recorded in a Microsoft Excel document for later processing.

### 2.4. Kinesthetic + Visual (Robot)

**Figure 2:**
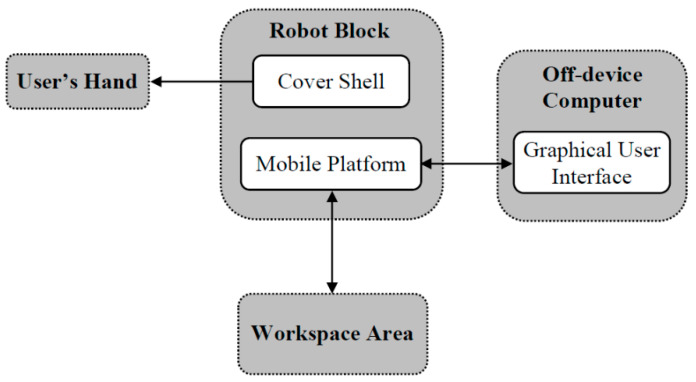
Block diagram illustrating the interaction between functional blocks of the robotic system.

A robotic system was created to act as a display for the program command sequence that can be followed both haptically and visually by the user. To do this, the user rested one of their hands on the cover shell of a palm-sized, tabletop mobile robot executing the program commands. The system was comprised of three functional blocks: (1) the robot block, consisting of a small, wheeled robot and a cover shell which directly interacted with a participant’s hand, (2) the planar workspace area on which the robot moved, and (3) the graphical user interface (GUI) used by the experimenter to control the robot. The interaction between the blocks is given in Figure 2.

#### 2.4.1. Robot Block

**Figure 3:**
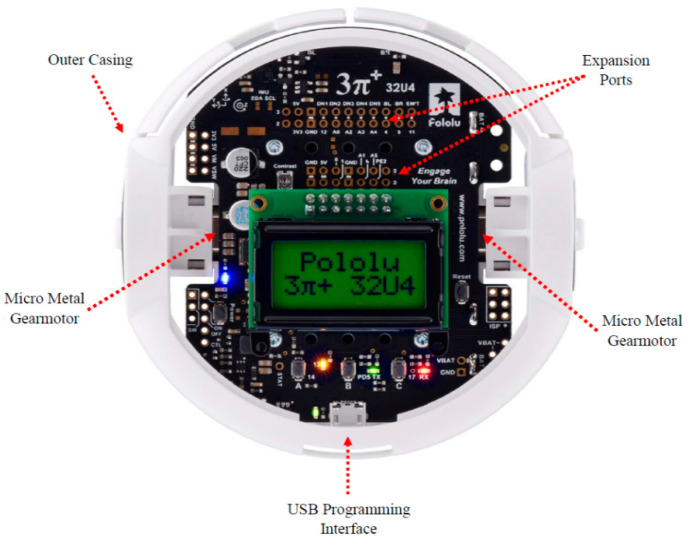
3pi+ 32U4 mobile platform [40].

The robot block consisted of a commercially available mobile platform (3pi+ 32U4—Turtle Edition, Pololu Corporation, Las Vegas, NV, USA), that acted as the central component of this system (Figure 3). This mobile platform was selected due to its ease of programming, miniature size, and high precision encoders allowing for greater movement accuracy than more commonly used mobile robots in education (e.g., Edison Robot, and Ozbots), some of which, at least, have extremely poor accuracy. Sufficient accuracy is needed for a programmer to ascertain if a program has done what they intended it to do, which, along with debugging, is a fundamental part of programming.

The platform is based on an ATmega32U4 AVR microcontroller (MCU) and is preloaded with an Arduino-compatible bootloader. A software add-on made it easy to program the platform from the Arduino IDE. The robot is equipped with two Micro Metal Gearmotors (with a miniature low-power, 6 V brushed DC motor with a 75.81:1 metal gearbox) placed concentrically 180° apart around the vertical axis of the platform. The miniature size (9.7 cm diameter) along with these gearmotors allows for a maximum platform speed of approximately 0.4 m/s. The dual quadrature wheel encoders included allow for closed-loop position control and provide a resolution of 12 counts per revolution of the motor shaft. Given the gear ratio and resolution, this mobile platform counts 909.72 ticks per wheel revolution. Since the wheel diameter is 32 mm, 909.72 ticks corresponds to a 100.48 mm linear distance (i.e., 0.1099 mm/tick). The platform is also equipped with a full IMU (with a LSM6DS33—3-axis accelerometer and a 3-axis gyroscope, and a LIS3MDL—3-axis magnetometer), which allows for the accurate control of the orientation with respect to the z-axis. The onboard MCU is responsible for receiving commands from the MATLAB^®^-based GUI, decoding these commands, and controlling the mobile platform. It is also responsible for relaying input from the device (measured position) to the underlying code of the GUI.

**Figure 4:**
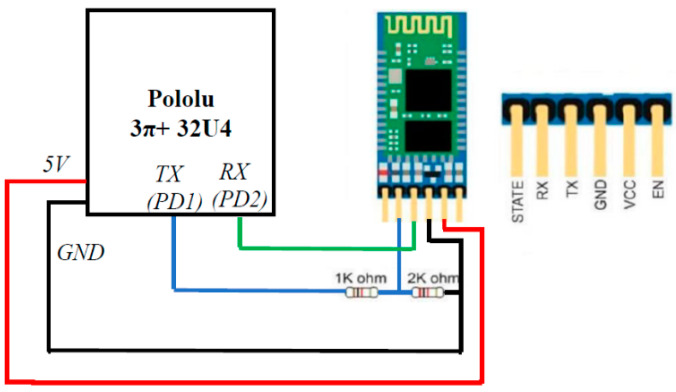
The diagram showing connections between the HC-05 module and the MCU.

We made several modifications to the mobile robot to make it more suitable for providing haptic feedback and the frequent need to modify and download code as the user learns through experience. To address this latter issue, wireless communication capability was added to the mobile platform by interfacing it to a HiLetgo HC-05 Bluetooth RF Transceiver over one of its expansion ports. The HC-05 uses serial communication to communicate with the MCU and Bluetooth to communicate with the computer creating the computer program. It can support data speeds up to 1 Mbps within the range of 10 m. One issue with the interface was that although the HC-05 module can be powered from 3.3 V to 6 V, it uses a 3.3 V logic level voltage, whereas the MCU supports a 5 V logic level. A logic level voltage divider was implemented to ensure safe communication between the module and the MCU. In addition, an external momentary push-on/push-off switch was added to help turn the robot on–off without having to remove the casing. Figure 4 shows the details of this setup.

**Figure 5:**
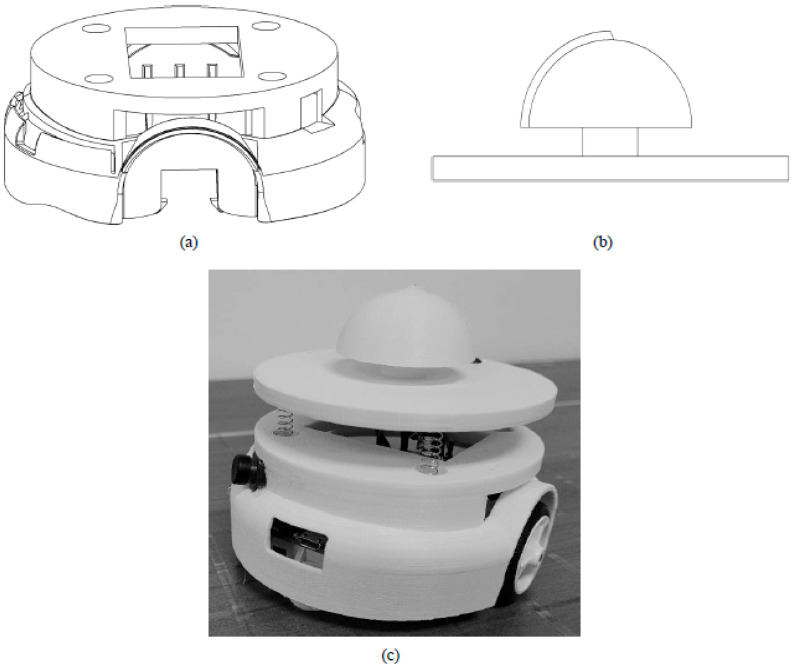
(**a**) Customized base; (**b**) customized top plate; (**c**) modified mobile platform.

To make the robot more suitable for providing haptic feedback, the outer casing of the mobile platform was replaced with the customized cover shell (Figure 5) to make it easier for a user to follow. The cover shell consisted of three components: a customized base (Figure 5a), a customized top (Figure 5b) and springs used to couple the two. The top was designed with a hemispherical crown for the palmar side of the hand to rest on and for allowing it to curve for a better grip. The ridge line was created to provide feedback as to the direction the robot was facing. The base was created to provide a secure mounting to the robot. Four evenly-spaced springs (with a length = 0.75 in and a spring constant = 2.17 lbs/in) were used to couple the base and top together. The springs were chosen to make the robot less susceptible to movement due to any force applied by the user.

#### 2.4.2. Graphical User Interface

**Figure 6:**
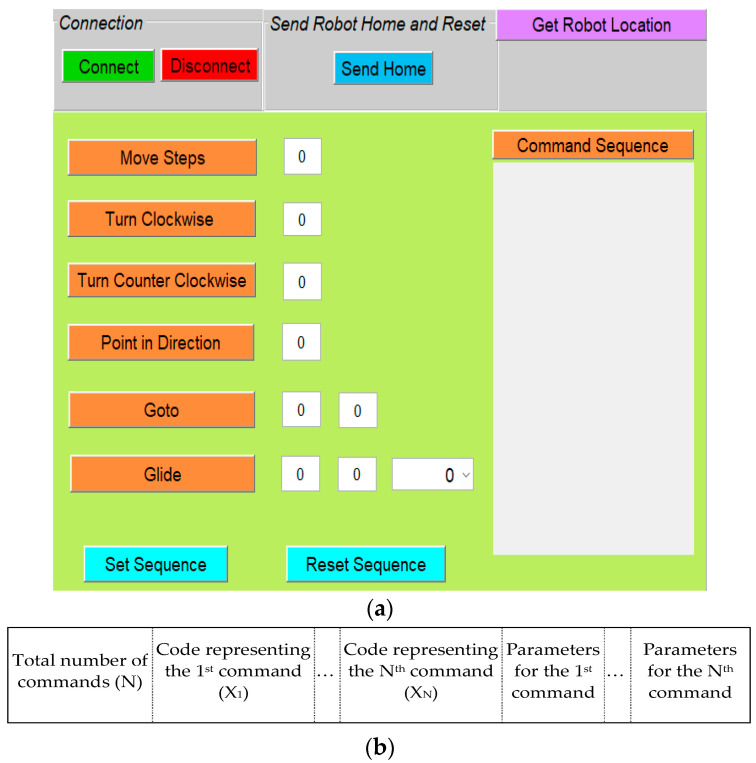
(**a**) The graphical user interface; (**b**) data packet format used for communication.

The graphical user interface (GUI) was created using MATLAB^®^ to facilitate a fast and easy implementation of the sequence of commands in a program (Figure 6a). Similar in function to the Scratch user interface, the MATLAB GUI allowed the experimenter to input the sequence of commands and their needed parameter values. To generate a program, once the parameters values were input in the middle section of the interface for the particular commands to be used, the experimenter could click on the commands’ buttons in any order to generate the program code sequence. The commands and corresponding parameter values were arranged in a data packet format (an array in MATLAB) shown in Figure 6b. The first cell in the data packet was used to store a number (N) that indicated the total number of commands in a program. The next N cells were used to store a code (X_N_) that represented a particular command. This was followed by the cells that stored the parameter values corresponding to each of the commands. To avoid any user input error in creating the program, the sequence of commands and corresponding data values were displayed in a display box named “command sequence”. The “set sequence” button was used to send the data packet (i.e., the commands and parameter values) to the mobile platform using Bluetooth communication. The commands then executed their corresponding function on the mobile robot that implemented the instruction.

#### 2.4.3. Workspace Area

**Figure 7:**
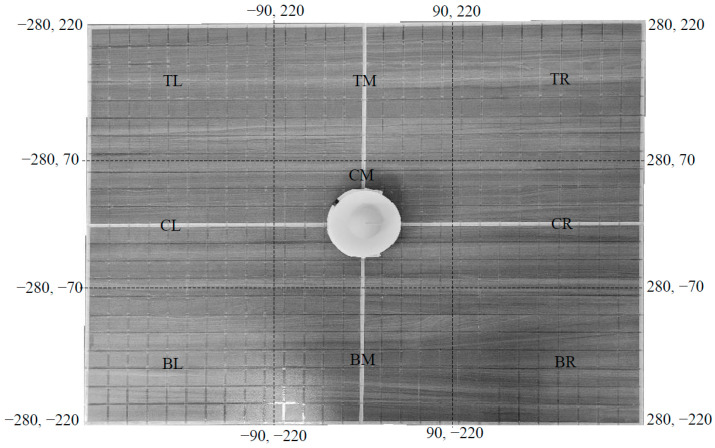
The workspace area of the robot. Grid lines are spaced one inch apart (both horizontally and vertically) and one inch of space corresponds to 20 points of movement on the Scratch workspace.

The workspace area of the robot consisted of a 28 in × 22 in (711.2 mm × 558.8 mm) tabletop space (Figure 7). This selection was influenced by two main factors. First, these dimensions always ensured that the robot was within arm’s reach of the participants (i.e., the participants could comfortably follow the robot over the entire space by resting their hand on the top). Second, the width-to-length ratio (1.27) of the robotic workspace corresponded closely to the width-to-length ratio of the Scratch (1.22), which kept the proportions of the two environments similar.

A tape grid was placed on the workspace to provide tactile feedback about the location (Figure 7). The workspace area was first divided into smaller, uniform squares of 1 in^2^ by placing grid lines of ¼” clear tape spaced every inch (25.4 mm) both horizontally and vertically. Pink 3/8” washi paper tape was then used to define the x and y axes and the border of the work area. The origin was defined by a ½” break in the washi tape defining the axes.

The starting planar position for the robot was in the center of the workspace (x = 0, y = 0). One inch of a linear robotic movement was represented as 20 points of movement on the Scratch workspace. The angular reference frame was also the same as in Scratch, starting at 0 degrees on the positive y-axis and increasing clockwise.

#### 2.4.4. Procedure

In this experimental condition, the participant sat in front of a workspace area (Figure 7) and rested one of their hands on the crown of the top plate on the mobile robot. The participants were instructed not to put too much hand pressure on the plate, just using this feature to allow their hand to follow the mobile robot. In addition, the participants were also instructed to keep their vision on the mobile robot throughout the movement period.

For each trial, the experimenter implemented the required sequence of commands using the GUI (Figure 6). To execute the program on the mobile robot, the experimenter pressed the “set sequence” button on the GUI. This sent the sequence of commands to the mobile platform over Bluetooth, resulting in the movement of the robot in the workspace. Similar to the other conditions, the participants were allowed to request a repetition of the mobile robot’s movements until they felt confident about their understanding of the program. Next, they were allowed only a single attempt to repeat a sequence of commands back to the experimenter. The user response information was recorded using a Microsoft Excel worksheet for later processing.

#### 2.4.5. Data Analysis

User response information (i.e., sequence of movement commands and planar positions) for the three feedback modalities (i.e., narrative, kinesthetic + visual, and visual only) was retrieved from the Microsoft Excel worksheet and processed using the statistical package for social sciences (SPSS). A repeated measure ANOVA (with the independent variable: feedback modality; dependent variable: mean accuracy across trials for that modality) was used to determine differences between the means of the three feedback modalities. If the effect was significant, post hoc matched paired *t*-tests were performed, using the Bonferroni correction for multiple comparisons. This analysis was performed for the mean accuracy of the identified commands and the mean accuracy of the identified zones of the end point at the end of each command. The mean accuracy of the identified commands for a given subject under a given condition was calculated as the total correctly-perceived program commands divided by the total number of program commands. The mean accuracy of the identified zones for a given subject under a given condition was calculated as the total number of times the end point zone of a command was determined as correctly-divided by the total number of times the end point zone was expected to be determined.

## 3. Results

**Figure 8:**
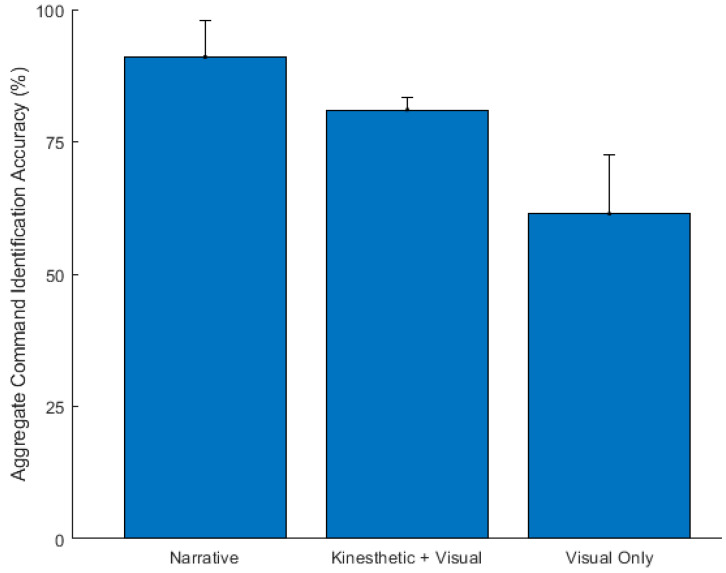
The aggregate command identification accuracy of the three feedback modalities across participants (N = 10).

As a starting point, we investigated the aggregate command (i.e., all commands lumped together) identification accuracy for each of the three feedback modalities. Figure 8 shows the mean (µ) and standard deviation (σ) of the command identification accuracy. The (µ ± σ) for participants using the narrative, kinesthetic + visual, and visual only modalities were: 91.0 ± 6.94, 81.0 ± 2.44, and 61.4 ± 11.12, respectively. Using a repeated-measures ANOVA, the identification accuracy differed significantly across the three modalities (F (2, 18) = 40.79, *p* < 0.001). A post hoc pairwise comparison, using the Bonferroni correction, showed that the identification accuracy with the narrative feedback was significantly higher than the kinesthetic + visual feedback (∆µ = 10, *p* = 0.004) and the visual only feedback (∆µ = 29.65, *p* < 0.001). Similarly, the identification accuracy with the kinesthetic + visual feedback was significantly higher than the visual only feedback (∆µ = 19.65, *p* < 0.001).

**Figure 9:**
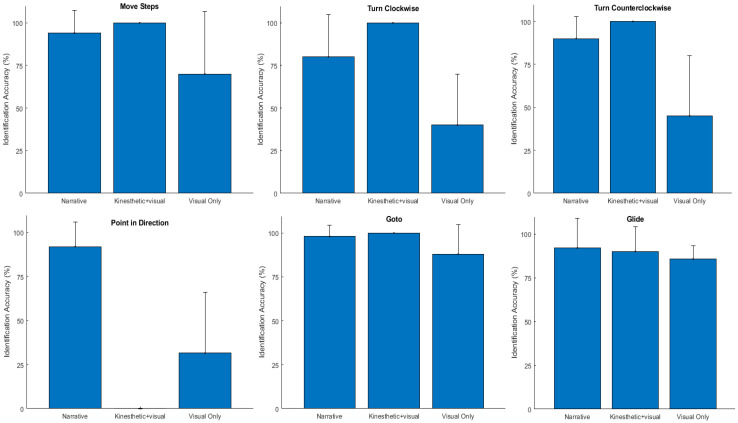
Individual movement command identification accuracy of the three feedback modalities across participants (N = 10).

To investigate this further, the command instruction identification accuracies for each of the six commands were compared for the three feedback modalities (i.e., narrative, kinesthetic + visual, visual only). Figure 9 shows the mean (µ) and standard deviation (σ) of the instruction identification accuracy for each command. A visual inspection indicated that the participants using the narrative and kinesthetic + visual feedback modalities had similar identification accuracies for all instructions except for the “point in direction”; however, the participants had difficulty identifying several of the commands using the visual only feedback modality. Table 4 presents the results of the statistical analysis for each individual instruction that paralleled that for the aggregate instructions. These results found that only one instruction, namely, the “point in direction”, had significantly different accuracy results between the narrative feedback and the kinesthetic + visual feedback. Comparing the visual only feedback condition to the other two, the results found significant differences between: (1) the visual only condition and the kinesthetic+ visual condition for the “turn clockwise instruction” and the “turn counterclockwise instruction, and (2) the visual only condition and the narrative condition for the “turn counterclockwise instruction” and the “point in direction instruction”.

**Figure 10:**
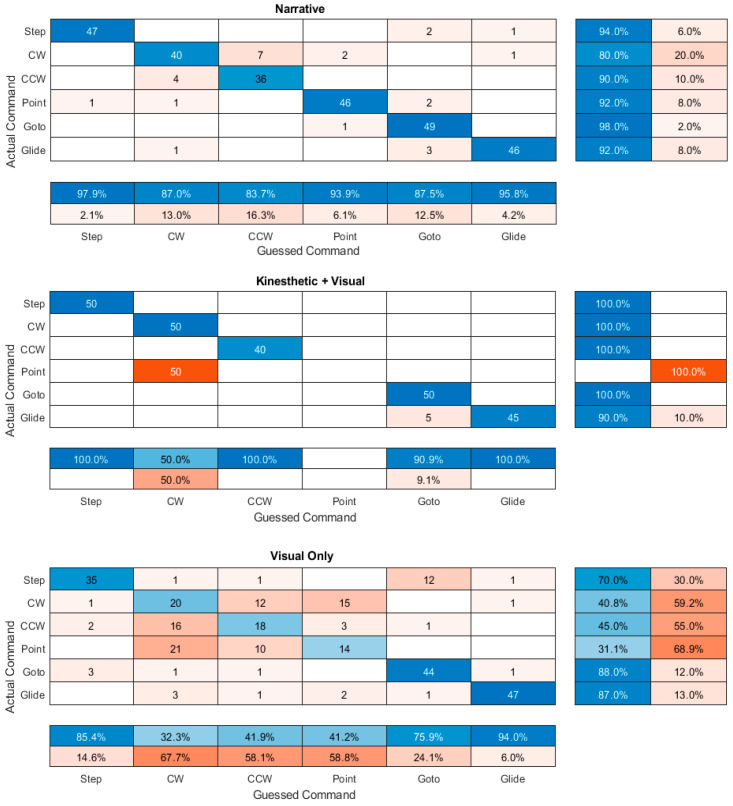
Confusion matrices. Each matrix shows the degree to which participants confused commands using the different feedback modalities. The diagonal elements represent the number for which the guessed (perceived) command is the same as the actual input command. Step: Move steps; CW: Turn clockwise; CCW: Turn counterclockwise; Point: Point in direction.

The participant confusion between the command instructions is given for each condition in Figure 10. Most of the inaccuracy in labeling the commands in the kinesthetic + visual feedback was due to the participants always labeling the point command as a clockwise rotation. Participant inaccuracy in the other feedback modalities was due to more widespread confusion. Much of the confusion was between motions of the same type: either rotational (CW, CCW, or Point) or translational (Step, Goto, or Glide); however, there were cases for both the narrative and visual only modalities where rotational motions were confused with translational motions.

To determine whether a program does what it is intended to do, it is also important for the creator to determine if they chose the parameter values correctly. For this to occur, it is first important that a robot is able to move accurately (Section 2.4.1). Moreover, it is also important that a programmer is able to determine the magnitude and direction of a motion accurately from the program’s execution. For this study, rather than asking for the parameter values (which vary for different commands), we asked the participants to provide the x, y coordinates of where the robot ended up after each command in a sequence. As participants in a pilot study found it very frustrating to provide the actual numeric x, y coordinates, the participants in the actual study were only asked to provide the location in terms of one of nine zones in the robot/cat’s workspace (Table 2).

**Figure 11:**
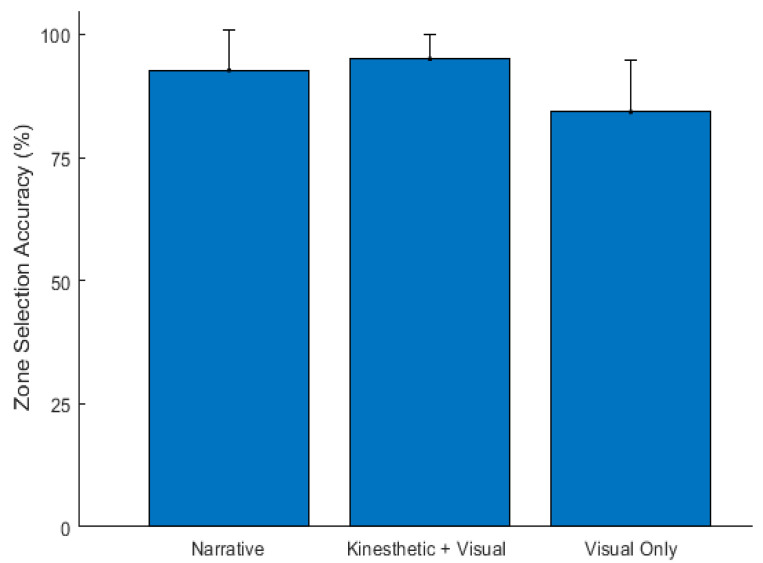
The zone selection accuracy of the three feedback modalities across participants (N = 10).

Figure 11 shows the mean (µ) and standard deviation (σ) of the zone selection accuracy (i.e., the end location accuracy) for the three feedback modalities. The mean and standard deviation (µ ± σ) for the participants using the narrative, kinesthetic + visual, and visual only modalities were 92.7 ± 8.17, 95.0 ± 5.15, and 84.2 ± 10.53, respectively. Using a repeated-measures ANOVA, the mean zone selection accuracy differed significantly across the three modalities (F(2, 18) = 6.03, *p* = 0.01). A post hoc pairwise comparison, using the Bonferroni correction, showed that the accuracy of the visual only modality was significantly lower than the kinesthetic + visual (∆µ = 10.73, *p* = 0.04) and narrative (∆µ = 8.47, *p* = 0.05) modalities, whereas there was no significant difference observed between the kinesthetic + visual and narrative modalities.

Participant perception of each of the methods used to illustrate the execution of a program was also important as, in a classroom or informal learning setting, this can affect a student’s willingness to persist in learning programing. The results for the questions asked in Table 3 are given in Table 5, where the positive and negative question results are combined to remove positive bias. This was calculated by: (value for positive question + (6 − value for negative question))/2.

Using a repeated-measures ANOVA, the participant response to the question pair 1/4 (ease of use) differed significantly across the three modalities (F(2, 18) = 6.16, *p* = 0.009). A post hoc pairwise comparison, using the Bonferroni correction, showed that the participants thought that using kinesthetic + visual feedback was easier to use compared to the narrative (∆µ = 1, *p* = 0.02) and visual only (∆µ = 0.75, *p* = 0.002) methods. Similarly, the participants thought that their confidence level in understanding the program (question pair 2/6) differed significantly across the three modalities (F(2, 18) = 4.99, *p* = 0.02). They also thought that using kinesthetic + visual feedback increased their confidence level significantly compared to using the narrative (∆µ = 0.95, *p* = 0.02) and visual only (∆µ = 0.1.05, *p* = 0.009) methods. In terms of program understanding (question pair 3/7), no statistical difference was found between the different feedback modalities (F(2,18) = 2.62, *p* = 0.10). Lastly, the participants also thought that the three feedback modalities differed in terms of how fun they were to use (question pair 5/8) (F(2,18) = 4.09, *p* = 0.03). A post hoc pairwise comparison revealed that the participants thought that using kinesthetic + visual feedback was much more fun to use compared to the narrative (∆µ = 0.7, *p* = 0.009) method. Although no statistically significant difference was found between using the kinesthetic + visual feedback and visual feedback only, there was a trend for the participants to perceive the kinesthetic + visual feedback as more fun.

When the participants were asked to rate the feedback modalities (i.e., 1st, 2nd, and 3rd preferences) in response to the questions asked in Table 6, 50% of them rated the kinesthetic + visual mode as their first priority for overall preference (Question 5, Table 6) whereas another 50% rated this mode as their second mode of choice. The participants had varying views about the other two modalities.

## 4. Discussion and Future Work

The use of kinesthetic feedback, in addition to the normally used visual feedback, may improve learning with educational robots by providing a richer, multi-modal experience that may appeal to a larger number of students with different learning styles; however, it is also possible that the addition of kinesthetic feedback, and how it may interfere with visual feedback, may decrease a student’s ability to interpret the program commands being executed by a robot. The ability to interpret the program commands being executed is critical for debugging, and is an important aspect of programming.

In this work, we investigated the potential for a tabletop robot that provided kinesthetic feedback, as well as visual feedback, about a program’s execution by facilitating contact with a user’s hand. Following the recommended educational practice, a student rested one of their hands on top of the robot and was able to pull it away at any time. The hand was also decoupled, to some extent, from the robot to minimize the influence of the student on the robot’s movement. This method was compared to the use of a visual only simulation and an audio narrative for presenting the program execution. The comparison was carried out in terms of the recall accuracy of the code command instruction and the planar position accuracy at the end of a command.

The initial results suggested that participants’ instruction recall accuracy using the narrative modality was statistically significant in having better accuracy than using the kinesthetic + visual and visual only modalities. Furthermore, using the kinesthetic + visual feedback performance was significantly better than using the visual only feedback modality; however, the confusion matrices (Figure 10) revealed that the only modality in which the participants did not confuse translational movements with rotational movements was the kinesthetic + visual condition. This was significant as errors were expected between some commands that could be hard to distinguish under certain conditions, such as the translational commands with each other and the rotational commands with each other. For example, a “goto” command could create the exact same movement as a “step” command if the x, y coordinates were directly in front of the robot; however, there was no possibility (except for no movement) where a translational movement could create the same motion as a rotational movement.

The recall errors of the command instructions used for the kinesthetic + visual modality were primarily due to the participants confusing the point command for clockwise rotation. In retrospect, this was not surprising as the point command was implemented by having the robot rotate clockwise. One could modify the implementation of the movements by changing the amount of time to rotate for a point command (e.g., fast) versus a clockwise rotation (e.g., slow). One could argue, however, that the true point at which the commands should be differentiated is when the code sequence is executed a second time from a different location: this is when, conceptually, a relative motion will be differentiated from a motion using absolute coordinates. Unfortunately, one weakness of the study was that the sequences of movements were not executed more than once without resetting the robot back to its initial point. A more problematic issue was that there was minor confusion between the “goto” and “glide” commands due, most likely, to the close similarity in the executed movement.

The largest recall errors of the command instructions using the narrative method were due to the confusion between clockwise movement and counterclockwise movement. On reflection, this is not surprising as the spoken words “clockwise” and “counter-clockwise” are probably the easiest to confuse of all the commands. This could be resolved by using alternate word descriptions for these commands, although it is difficult to conceive a description that is not ambiguous in meaning (as opposed to sounds) and that is still brief.

The participants had many more errors when using the visual only feedback compared to the other two. A significant portion of these errors was due to confusion between the commands in absolute coordinates and relative coordinates that, based strictly on the motions, should not be disambiguated until the code sequence is executed a second time from a new location. This confusion does pose some difficulty in learning to program with both the visual only feedback and the kinesthetic/haptic + visual feedback, as the commands are not obvious immediately during execution; however, the addition of further feedback (such as audio) may introduce other problems as one is also able to program the audio output in Scratch, and the code executed concurrently with the motions. There also may be some instructional value to teaching students the algorithmic difference between relative movements and ones that use absolute coordinates. The actual movement could be interpreted correctly as a relative or an absolute command. It is possible that the relative command was more commonly (in fact, exclusively) used because it is more akin to the use of egocentric spatial reference frames (with the participant imagining themselves as the robot), which are more inherent in human neural representations than allocentric (absolute) reference frames [41].

A notable additional concern with using the visual only method, implemented by using the online Scratch environment “stage”, was that there was significant confusion between clockwise and counterclockwise rotation, even though, conceptually, these are very different motions. This was likely due to the environment’s implementation of all motions, except “glide”, which occurred instantaneously; thus, clockwise and counterclockwise produced a similar animation. This could be resolved by using a more realistic animation, but it is not clear what other problems this would create in the environment. This is also not the only consideration when considering a visual only versus a kinesthetic+ visual feedback.

Another important aspect of determining whether a program is working is validating that the magnitude of movement, set by the parameters in the program, are correct. The zone accuracy of the participants was significantly lower using the visual only feedback as compared to the kinesthetic + visual and narrative feedback modalities. Neither of the latter two modalities were found to be significantly different from each other. This suggests that the latter two modalities might be more helpful to participants in the debugging process. One limitation of the study is that we asked the participants to provide the spatial zone that the robot/cat was in rather than the actual x, y coordinates, which would have provided a more precise measure of accuracy. It would also have been beneficial to ask the participants for the direction that the robot/cat was facing; however, the participants already felt overloaded by what they needed to remember.

In addition to the aforementioned limitations, one other limitation was the use of only two wheels for the physical robot. This resulted in the movement commands changing conceptually for the “goto” and “glide” commands: to execute these commands the robot needed to rotate to face the location it needed to move to, move to that location, and then reorient itself to its original direction. Surprisingly, there was never an instance where the participants misinterpreted one of these commands with a sequence of three commands (i.e., rotate, move, and rotate). This issue could be resolved, though, by using an omni-wheeled robot [42] which can move in any direction without changing orientation.

Finally, the post-study survey results suggest that the participants thought that the kinesthetic + visual mode was easy and fun to use and increased their confidence level in terms of understanding the program. Moreover, when given a choice, they preferred this feedback mode over the narrative and visual only modes. This aligns with the results in previous literature that suggests that most engineering students prefer multi-modal feedback compared to a single modality. Although the difference in perceived fun was not statistically significant between the kinesthetic + visual mode and the visual only mode, this may have been due to the relatively small number of participants in the study.

This paper showed that individuals were able to accurately determine a sequence of movement commands and their magnitude when using combined kinesthetic + visual feedback. Overall, these results indicate that the robotic method (i.e., using kinesthetic + visual feedback) can be used as a practical and effective method to perceive program execution. This is especially important because although similar results were obtained for the narrative feedback, it is not a very practical solution in a classroom with multiple students. Moreover, the participants ranked the narrative method as their least-preferred method. Additionally, knowing that adding kinesthetic feedback to visual feedback does not produce any adverse effects in perception, future work can now study the impact of adding kinesthetic feedback on student learning.

## Figures and Tables

**Table 1:** List of instructions and parameters.

Instruction	Function
Move # Steps	Move forward (±) # steps from the current location
Turn Clockwise #	Rotate in a clockwise direction # degrees from the current direction faced
Turn Counterclockwise #	Rotate in a counterclockwise direction # degrees from the current direction faced
Point in Direction #	Point in a specific direction # degrees on a Cartesian coordinate system irrespective of the current orientation
Go to #, #	Move to a particular planar location (±x and ±y) in a Cartesian coordinate system
Glide #, #, #	Move to a particular planar location (±x and ±y) in a Cartesian coordinate system, taking t seconds to complete the movement

**Table 2:** The planar position and the corresponding zone.

Position	Zone
x < −90 and y > 70	Top Left (TL)
−90 >= x <= 90 and y > 70	Top Middle (TM)
x > 90 and y > 70	Top Right (TR)
x < −90 and −70 >= y <= 70	Center Left (CL)
−90 >= x <= 90 and −70 >= y <= 70	Center Middle (CM)
x > 90 and −70 >= y <= 70	Center Right (CR)
x < −90 and y < −70	Bottom Left (BL)
−90 >= x <= 90 and y < −70	Bottom Middle (BM)
x > 90 and y < −70	Bottom Right (BR)

**Table 3:** List of questions for each feedback modality.

Question Number	Statement
1	I thought this method was easy to use
2	I did not feel very confident using this method
3	I thought this method helped me understand the program quickly
4	I thought this method was very cumbersome to use
5	I thought this method was fun to use
6	I felt very confident using this method
7	I thought this method was not very helpful in understanding the program
8	I thought this method was boring to use

**Table 4:** Comparison of movement command identification accuracy for individual instructions (where for each participant the mean value of a particular correctly-perceived command was used as a single data point): mean (µ) and standard deviation (σ) for each modality; the outcome of comparing the feedback modalities using repeated measures ANOVA; and the outcomes of the matched paired *t*-tests, using the Bonferroni adjustment, for the statistically significant differences, with the pairs indicated by * narrative ≠ kinesthetic + visual; ** narrative ≠ visual only; *** kinesthetic + visual ≠ visual only.

	Narrative (µ ± σ)	Kinesthetic + Visual (µ ± σ)	Visual Only (µ ± σ)	ANOVA (F, *p*)	Bonferroni Pairwise Comparison
Move Steps	94 ± 13.50	100 ± 0	70 ± 36.82	F(2, 18) = 4.71, *p* = 0.023	
Turn Clockwise	80 ± 24.94	100 ± 0	40 ± 29.81	F(2, 18) = 13.70, *p* < 0.001	*** (∆µ = 60, *p* < 0.001)
Turn Counterclockwise	90.0 ± 12.91	100 ± 0	45.0 ± 34.96	F(2, 18) = 15.58, *p* < 0.001	** (∆µ = 45, *p* = 0.03) *** (∆µ = 55, *p* = 0.002)
Point in Direction	92 ± 13.98	0 ± 0	31.5 ± 36.65	F(2, 18) = 58.77, *p* < 0.001	* (∆µ = 92, *p* < 0.001) ** (∆µ = 60.5, *p* < 0.001)
Goto	98 ± 6.33	100 ± 0	88 ± 16.87	F(2, 18) = 4.04, *p* = 0.04	
Glide	95.6 ± 13.33	88.9 ± 14.53	85.9 ± 8.13	F(2, 16) = 1.80, *p* = 0.20	

**Table 5:** Participant responses (positive and negative responses combined) to the questions from Table 3.

Question Number	Narrative (µ ± σ)	Kinesthetic + Visual (µ ± σ)	Visual Only (µ ± σ)
1/4 (Ease of Use)	2.85 ± 1.11	3.85 ± 0.75	3.10 ± 0.70
2/6 (Confidence Level)	2.45 ± 1.17	3.40 ± 0.77	2.35 ± 1.29
3/7 (Understanding)	3.33 ± 0.92	3.75 ± 0.49	3.45 ± 0.83
5/8 (Fun to Use)	3.35 ± 0.85	4.05 ± 0.55	3.45 ± 1.09

**Table 6:** List for ranking feedback modes based on preference.

Question Number	Statement
1	The ease of use for understanding a program
2	Your confidence in understanding a program
3	The speed of understanding a program
4	How fun it was for understanding a program
5	Your overall preference

## Data Availability

Please contact Dianne T. V. Pawluk via email to obtain data.
